# The Influence of a KDT501, a Novel Isohumulone, on Adipocyte Function in Humans

**DOI:** 10.3389/fendo.2017.00255

**Published:** 2017-09-29

**Authors:** Brian S. Finlin, Beibei Zhu, Bernard P. Kok, Cristina Godio, Philip M. Westgate, Neile Grayson, Robert Sims, Jeffrey S. Bland, Enrique Saez, Philip A. Kern

**Affiliations:** ^1^The Department of Internal Medicine, Division of Endocrinology, The Barnstable Brown Diabetes and Obesity Center, University of Kentucky, Lexington, KY, United States; ^2^Department of Molecular Medicine, Scripps Research Institute, La Jolla, CA, United States; ^3^College of Public Health, University of Kentucky, Lexington, KY, United States; ^4^Kindex Pharmaceuticals, Seattle, WA, United States

**Keywords:** adiponectin, adipocyte secretion, adipose tissue biology, gene expression profiling, metabolic syndrome, mitochondria

## Abstract

**Objective:**

In a phase II clinical trial in nine obese, insulin-resistant humans, we observed that treatment with KDT501, a novel isohumulone drug, increased total and high-molecular weight (HMW) adiponectin in plasma. The objective was to determine whether KDT501 increased adiponectin secretion from subcutaneous white adipose tissue (SC WAT) and the underlying mechanism(s).

**Methods:**

Nine obese participants with either prediabetes or with normal glucose tolerance plus three features of metabolic syndrome were part of the study. SC WAT biopsies were performed before and after 28 days of KDT501 treatment in a clinical research setting. In addition, a cold stimulus was used to induce thermogenic gene expression. Adiponectin secretion was measured, and gene expression of 130 genes involved in adipose tissue function was determined. The effect of KDT501 on adipocyte mitochondrial function was analyzed *in vitro*.

**Results:**

SC WAT explants secreted more total and HMW adiponectin after KDT501 treatment (*P* < 0.05). After KDT501 treatment, a number of genes involved in thermogenesis and lipolysis were induced by cold (*P* < 0.05). KDT501 also potentiated β-adrenergic signaling (*P* < 0.001) and enhanced mitochondrial function in adipocytes (*P* < 0.001).

**Conclusion:**

KDT501 induced adiponectin secretion posttranscriptionally and increased gene expression of thermogenic and lipolytic genes in response to cold stimulation. These beneficial effects on SC WAT may be explained by the ability of KDT501 to potentiate β-adrenergic signaling and enhance mitochondrial function in adipocytes.

**Clinical Trial Registration:**

https://www.ClinicalTrials.gov, ID number: NCT02444910.

## Introduction

KDT501 is a compound chemically derived from hops that has antidiabetic effects in rodents (1). More specifically, KDT501 is the potassium salt of the *n*-(isobutyl) congener of a tetrahydro iso-alpha acid, also known as isohumulone. We have recently analyzed the effect of 1 month of KDT501 treatment in nine obese, insulin-resistant subjects in an open-label, phase II clinical trial (2). Although oral glucose tolerance did not improve significantly, there were improvements in several secondary outcomes. KDT501 treatment reduced plasma triglyceride levels during a lipid tolerance test. Total and high-molecular weight (HMW) adiponectin were higher in plasma after KDT501 treatment (2). Adiponectin is an anti-inflammatory, insulin-sensitizing adipokine that is negatively correlated with several aspects of metabolic syndrome and polycystic ovary syndrome (PCOS) (3) and acts at multiple tissues to promote metabolic homeostasis [recently reviewed in Ref. (4)]. Consistent with this, plasma TNFα was reduced by KDT501 treatment (2).

Adiponectin is specifically expressed by adipocytes, suggesting that KDT501 treatment in this clinical trial affected adipose tissue to increase plasma adiponectin levels (2). KDT501 has pleiotropic effects on gene expression *in vitro*. For instance, KDT501 promoted adiponectin gene expression in a human adipocyte primary cell culture model (1). KDT501 also inhibited inflammatory gene expression in macrophages *in vitro* (1). In obesity, adipose tissue secretes TNFα (5, 6), an inflammatory cytokine that is inversely coordinated with adiponectin expression and insulin sensitivity (7). These observations suggest that KDT501 treatment reduces some measures of adipose dysfunction in part by remodeling gene transcription.

In this study, we focused on adipose tissue to identify mechanisms by which KDT501 functions *in vivo* in these nine obese human research participants. We evaluated adiponectin secretion from abdominal subcutaneous white adipose tissue (SC WAT) explant secretions of the participants before and after treatment with KDT501 from our phase 2 study (2). We then comprehensively evaluated the subcutaneous adipose tissue transcriptional response to KDT501. Since KDT501 promotes weight loss in rodents (1), we hypothesized that KDT501 treatment would reduce adipose dysfunction that occurs with obesity in humans. Comprehensive analysis of gene expression could thus provide insight into the mechanism by which KDT501 increases adiponectin secretion *in vivo* and affects overall SC WAT function. The panel of genes in our multiplex assay included genes involved in the function of adipose tissue and genes involved in adipose dysfunction such as inflammatory cytokines, immune cell type markers, and fibrosis genes. Because KDT501 was found to stimulate thermogenic genes in cultured human adipocytes (1), we measured these genes before and after a brief cold stimulus. To gain mechanistic insight into the effect of KDT501 on adipocytes, we evaluated the effect of KDT501 on adipocyte mitochondrial bioenergetics and fatty acid oxidation *in vitro*.

## Materials and Methods

### Human Subjects and Study Design

All subjects gave informed consent, and the protocols were approved by the Institutional Review Board at the University of Kentucky. Recruitment for this study was through local advertising and initial phone screening eliminated from consideration participants who could not participate or who did not meet inclusion criteria, such as BMI and age. 15 potential subjects were invited for in-person screening, which included an OGTT and fasting labs, and 9 of these subjects met inclusion/exclusion criteria and were enrolled in the study. All nine of these subjects completed the study. The characteristics of these subjects; inclusion and exclusion criteria; and the Phase II KDT501 drug trial (ClinicalTrials.gov ID number NCT02444910) have recently been reported (2). In brief, nine obese, non-diabetic, insulin-resistant subjects were given KDT501 in escalating doses for 28 days during the summer. The relatively short duration of treatment and the limited doses of KDT501 was a requirement of the FDA during this phase 2A study. This short-term treatment did not cause a change in appetite, weight (pre: 100.8 ± 5.5 kg; post: 100.3 ± 5.7 kg; *P* = 0.63), % body fat (pre: 47.5 ± 1.55; post: 47.6 ± 1.5; *P* = 0.5), resting energy expenditure (pre: 1,725 ± 79 kcal/day; post: 1,670 ± 106 kcal/day; *P* = 0.29), or respiratory quotient (pre: 0.86 ± 0.02; post: 0.87 ± 0.02; *P* = 0.0.86); all values are mean ± SEM. SC WAT biopsies were performed before and after 28 days of KDT501 treatment; all posttreatment biopsies were obtained between 0 and 3 days after the last dose of KDT501. In addition, a cold stimulus was used to determine whether KDT501 affected thermogenic gene induction. A subcutaneous adipose biopsy was performed on the right side of the abdomen. An ice pack was applied to the left side of the abdomen for 30 min, and a second biopsy was performed 4 h later on the cold-stimulated adipose. A portion of the adipose tissue was analyzed for secretion as described below. The remaining adipose tissue was immediately frozen in liquid nitrogen and stored at −80°C.

### Adipose Tissue Explant Secretions

We weighed 0.5 g of fat from each SC WAT biopsy, placed the explant into 2 mL Dulbecco’s modified Eagles’ medium (DMEM), and incubated for 1 h at 37°C. The adipose explant was removed, and the DMEM was centrifuged to remove debris. The experiment thus maintained the SC WAT weight to volume of DMEM ratio similar to the normalization applied by Kovacova and colleagues (8). The medium was diluted 1:100 and analyzed with a total and HMW adiponectin ELISA kit (47-ADPHU-E01; Alpco, Salem, NH, USA) according to the manufacturer’s instructions.

### Gene Expression

RNA was prepared from the subcutaneous adipose tissue using a Qiagen RNAeasy Lipid Tissue Mini kit (Qiagen, Valencia, CA, USA). RNA quantity and quality were determined using an Agilent 2100 Bioanalyzer (Palo Alto, CA, USA). The expression of 130 genes was analyzed using the Nanostring nCounter multiplex system (gene names and accession numbers are in Table S1 in Supplementary Material). The data were normalized to the positive controls and the geometric mean of six housekeeping genes (Table S1 in Supplementary Material) according to the manufacturer’s instructions. The background level of the standard negative controls supplied by Nanostring was very low and not subtracted. We also determined the negative control value of the individual probes by doing no RNA (water) controls. These values, which are the average of three controls, were also low (see Tables S2–S5 in Supplementary Material). The gene expression of ERp44, ERo1-La, and DsbA-L was determined by real-time reverse transcriptase polymerase chain reaction (RT-PCR) as described (9) and normalized the geometric mean of the same six genes utilized in the Nanostring assay (Table S1 in Supplementary Material).

### Cell Culture

Primary brown preadipocytes were isolated from neonatal wild-type C57BL/6 pups as previously described (10). Briefly, brown adipose tissue was digested for 30 min at 37°C using 1.5 mg/mL collagenase type I (Worthington) in PBS containing the following additional components: 62 mM NaCl, 2.5 mM KCl, 0.65 mM CaCl_2_, 2.5 mM glucose, 50 mM HEPES, and 2% fatty acid-free BSA. Cells were strained using a 100 µM filter, plated, and grown to confluence in 20% FBS-DMEM. Differentiation was initiated by replacing the growth media with 10% FBS-DMEM supplemented with 172 nM insulin, 1 µM dexamethasone, 0.5 mM 3-isobutyl-1-methylxanthine (IBMX), 1 nM triiodothyronine (T3), and 1 µM rosiglitazone. After 2 days, adipogenesis induction media were replaced with 10% FBS-DMEM containing 172 nM insulin and 1 nM T3 and refreshed every other day. Five days after the induction of differentiation, differentiated brown adipocytes were replated onto a 96-well Seahorse plate at a density of 5,000 cell/well and allowed to settle overnight. Cells were treated with vehicle, rosiglitazone, and KDT501 for 16 h before analysis of mitochondrial respiration parameters on a Seahorse XFe96 extracellular flux analyzer. 3T3-L1 preadipocytes were cultured and differentiated as previously described (11). Briefly, 3T3-L1 preadipocytes were maintained in 10% BCS-DMEM. To induce differentiation, cells were replated and allowed to reach confluence. Two days post-confluence, cells were treated with 172 nM insulin, 1 µM dexamethasone, 0.5 mM IBMX, and 1 µM rosiglitazone in 10% FBS-DMEM. After 2 days, adipogenesis induction media were replaced with 10% FBS-DMEM supplemented with 172 nM insulin. Differentiated adipocytes were replated onto 96-well Seahorse plates at 6,000 cells per well and allowed to settle overnight. Cells were treated with vehicle, rosiglitazone, or KDT501 for 48 h before the fatty acid oxidation rate was measured in the Seahorse XFe96.

### Mitochondrial Bioenergetics and Fatty Acid Oxidation Rate Assays

Analyses of mitochondrial respiration parameters and fatty acid oxidation rates were performed in an XFe6 instrument following the manufacturer’s instructions (Seahorse Bioscience). Briefly, primary brown adipocytes were switched to substrate-free DMEM supplemented with 10 mM glucose, 10 mM pyruvate, 1× Glutamax, 10 mM HEPES pH 7.4, and a Mitostress test was performed. The oxygen consumption rate was measured in real time to document basal respiration and four sequential injections were then performed: (1) 100 nM norepinephrine (NE); (2) 2 µM oligomycin; (3) 1 µM carbonyl cyanide-4-(trifluoromethoxy)phenylhydrazone (FCCP); and (4) a combination of 2 µM rotenone and 2 µM antimycin A. To measure the fatty acid oxidation rate, 3T3-L1 adipocytes were switched to assay media (Krebs–Henseleit Buffer, 2.5 mM final glucose, 0.5 mM carnitine, and 5 mM HEPES; pH 7.4) for 1 h before the addition of vehicle (33 µM BSA) or 167 µM palmitate in complex with BSA (5:1). Real-time oxygen consumption was measured immediately after palmitate addition and following injections of 4 µM oligomycin, 4 µM FCCP, and a combination of 2 µM rotenone and 2 µM antimycin A. The concentration of oligomycin and FCCP was increased in fatty acid oxidation rate assays to account for the presence of BSA.

### Statistics

Unless noted, data were analyzed with a two-tailed, paired *t*-test. To identify genes that had a differential response to cold stimulus by KDT501 treatment, we calculated the difference in the response to cold stimulus before and after KDT501 treatment and then performed a two-sample *t*-test on this difference. Statistical significance was set at *P* < 0.05. Data from *in vitro* experiments with cultured adipocytes were analyzed by one-way analysis of variance (ANOVA).

## Results

### KDT501 Treatment Stimulates Adiponectin Secretion

**Figure 1:**
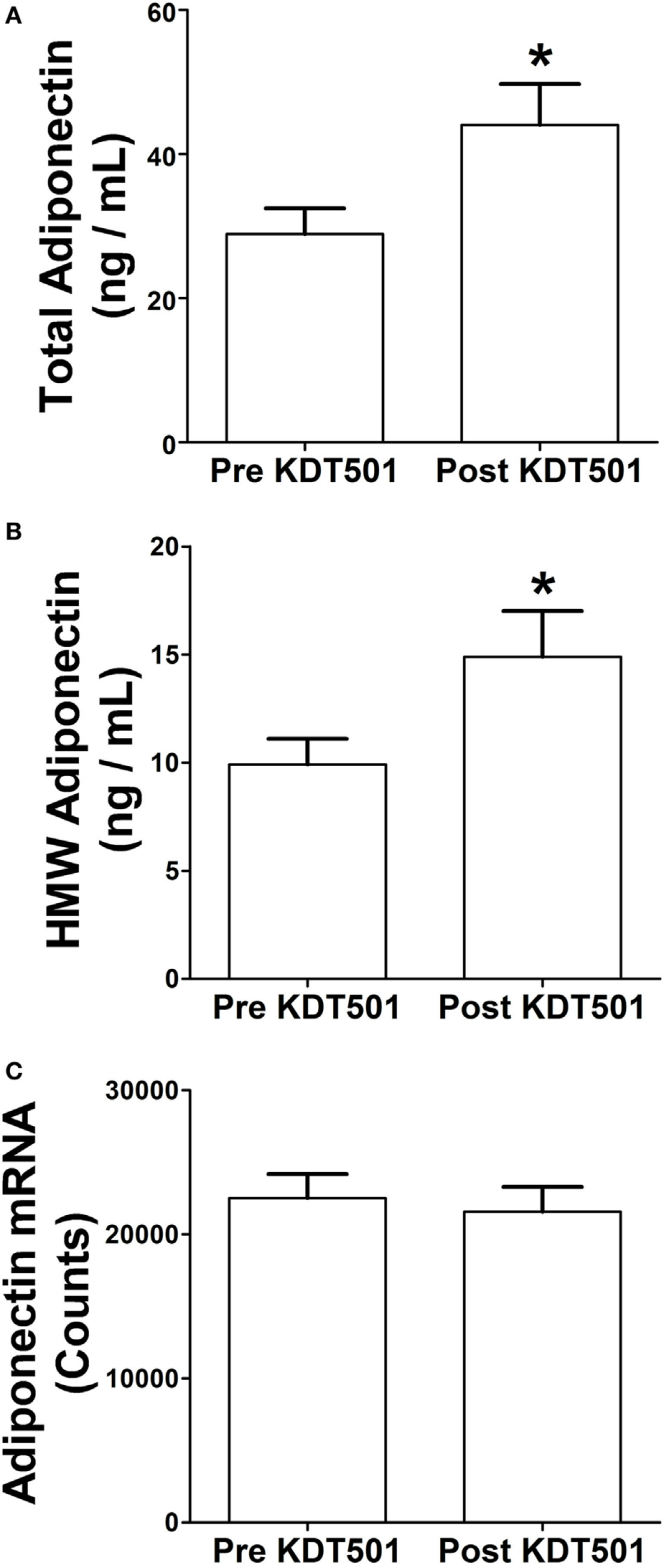
KDT501 induces total and high-molecular weight (HMW) adiponectin secretion by adipose tissue explants from obese, insulin-resistant subjects. Adipose tissue explants obtained before and after KDT501 treatment were incubated in Dulbecco’s modified Eagles’ medium (DMEM) for 1 h at 37°C. **(A)** Total and **(B)** HMW adiponectin concentrations in the DMEM were measured by ELISA and are expressed as the concentration/g adipose/h. **(C)** Adiponectin gene expression was measured in the corresponding adipose tissue biopsy with the Nanostring nCounter system as described in the Section “Materials and Methods.” Data represent the mean ± SEM (*n* = 9); data in all panels were analyzed by a paired, two-tailed Student’s *t*-test (*P < 0.05).

We previously reported that KDT501 treatment increased total and HMW adiponectin levels in plasma (2). When the SC WAT biopsies were performed in that study, a portion was immediately frozen for mRNA analyses and another portion (0.5 g) was placed in DMEM for 1 h so that we could analyze secreted proteins. We evaluated total and HMW adiponectin secretion from SC WAT explants before and after KDT501 treatment. KDT501 treatment induced a 1.5-fold increase in the secretion of total adiponectin (Figure 1A; *P* < 0.05) and HMW adiponectin (Figure 1B; *P* < 0.05) from the adipose tissue explants. However, adiponectin gene expression was unchanged by KDT501 treatment (Figure 1C), indicating that the increase in secretion occurs by a posttranscriptional mechanism. These results indicate that increased secretion of adiponectin from subcutaneous adipose tissue contributes to the increase in plasma adiponectin of these research participants after KDT501 treatment (2). Linear regression modeling was used to assess associations between changes in adiponectin secretion by KDT501 treatment and gender, age, and BMI. However, results were not statistically significant and therefore are not presented.

### Effect of KDT501 on Adipose Tissue Gene Expression

To identify potential mechanisms of action of KDT501, we analyzed gene expression in abdominal SC WAT with the Nanostring nCounter multiplex system. The gene panel comprised cytokines, chemotactic factors, immune cell markers, fibrosis markers, angiogenesis markers, genes involved in lipid handling, adipogenesis makers, genes involved in thermogenesis, and markers of brown and beige adipose (Table S1 in Supplementary Material). Table 1 shows that KDT501 treatment caused significant changes in only four adipose genes; four additional genes with non-significant trends (0.05 < *P* < 0.1) for change are also indicated in Table 1. KDT501 treatment reduced the mRNA expression of ACACA (Table 1; 0.86-fold; *P* = 0.038), which regulates fatty acid synthesis, and DGAT, which regulates triglyceride formation (Table 1; 0.87-fold; *P* = 0.043). The mRNA expression of LPL, which regulates lipid and lipoprotein uptake, tended to be reduced (Table 1; *P* = 0.068). Together, these results suggest a slight reduction in genes controlling adipose tissue lipid synthesis, uptake, and storage. MPZL2, which is a brown adipose marker (12), was induced 1.4-fold (Table 1; *P* = 0.041), and there was a trend for an increase in DIO2, which is involved in energy metabolism through the conversion of T4 into T3. However, UCP1 (*P* = 0.7) and PGC1α (*P* = 0.37), genes that are characteristic of brown and beige adipocytes, were not changed (Table S2 in Supplementary Material). The significance of the increases in MPZL2 and TNFRSF12A (Fn14, which is involved in many cell processes, especially involving tissue injury) is not known. Although KDT501 has anti-inflammatory effects *in vitro*, KDT501 treatment did not significantly reduce TNFα, IL12β, IL1β, or other inflammatory cytokines and immune cell markers in the multiplex assay panel (Table S2 in Supplementary Material). Finally, KDT501 did not change leptin gene expression (Table S2 in Supplementary Material). The data for all of the genes measured and the negative control values for each probe are in Table S2 in Supplementary Material.

**Table 1:** Gene expression changed by KDT501 treatment.

Gene^a^	Pre KDT501	Post KDT501	Fold change	*P*
			Post/pre	
TNFRSF12A	12 ± 2	16 ± 2	1.345	0.037
ACACA	229 ± 45	196 ± 35	0.857	0.038
MPZL2	52 ± 8	73 ± 12	1.407	0.041
DGAT2	12,059 ± 2,187	10,482 ± 1,941	0.869	0.043
LPL	17,455 ± 2,836	15,793 ± 2,584	0.905	0.068
TMEM26	16 ± 2	13 ± 2	0.856	0.072
TIMP1	1,382 ± 89	1,600 ± 158	1.157	0.075
DIO2	27 ± 6	38 ± 9	1.392	0.095

*^a^SCWAT was isolated before and after KDT501 treatment, and gene expression determined with the Nanostring nCounter system as described in the Section “Materials and Methods”*.

*The data are presented as means (nCounter Counts) ± SEM (*n* = 9; two-tailed, paired Student’s *t*-test)*.

### KDT501 Treatment Alters the Response of Adipose Tissue to Cold Stimulation

KDT501 promotes weight loss in mice, and microarray results from human adipocytes indicate that KDT501 stimulates thermogenic pathways in adipocytes (1), suggesting that KDT501 may promote WAT beiging. We have previously reported that a 30-min cold exposure stimulates beiging pathways in the adipose tissue of lean humans (13). Therefore, before and after KDT501 treatment, we applied an ice pack to one side of the abdomen for 30 min and 4 h later performed an adipose tissue biopsy, as described in the Section “Materials and Methods.” We then determined whether this cold stimulus changed gene expression and whether KDT501 treatment altered the response to cold. Before KDT501 treatment, the application of cold resulted in the significant repression of four unrelated genes, SCD, MMP14, AGTR2, and IL18 (Table 2; data for all of the remaining genes are in Table S3 in Supplementary Material).

**Table 2:** Gene expression changed by cold before KDT501 treatment.

Gene^a^	Pre cold	Post cold	Fold change	*P*
			Post/pre	
SCD	49,210 ± 12,496	44,454 ± 12,115	0.90	0.008
AGTR2	7 ± 1	4 ± 1	0.66	0.013
MMP14	548 ± 40	499 ± 34	0.91	0.030
IL18	88 ± 10	74 ± 5	0.83	0.034
FABP1	10 ± 2	5 ± 1	0.51	0.055
ACLY	2,998 ± 624	2,490 ± 444	0.83	0.061
ADRB2	72 ± 4	66 ± 4	0.92	0.066
CD163	1,074 ± 84	978 ± 71	0.91	0.069
THBS1	968 ± 125	1,163 ± 149	1.20	0.079
RBP4	23,669 ± 1,752	21,269 ± 2,232	0.90	0.091
CCL24	90 ± 21	75 ± 16	0.83	0.094
ADIPOQ	22,520 ± 1,671	20,773 ± 1,250	0.92	0.100

*^a^SCWAT was isolated before and after the cold stimulus before KDT501 treatment, and gene expression determined with the Nanostring nCounter system as described in the Section “Materials and Methods”*.

*The data are presented as means (nCounter Counts) ± SEM (*n* = 9; two-tailed, paired Student’s *t*-test)*.

After KDT501 treatment, a number of genes involved in lipolysis and lipid catabolism were significantly induced by cold (Table 3). These included PPARα, PGC1α, HSL, and ANGPTL4 (Table 3). Adipose triglyceride lipase (PNPLA2) and the coactivator of ATGL (CGI-58; ABHD5) showed a trend for induction (Table 3). VEGF, which stimulates angiogenesis and beiging (14), was also induced by icing (Table 3). Despite the induction of lipolytic genes and PGC1α by cold, the 1.2-fold increase in UCP1 was not significant (*P* = 0.34; Table S4 in Supplementary Material). CCL1, a chemokine for eosinophils and Th2 T cells (15, 16), was induced by cold after KDT501, which could be important given the role of type 2 immunity (e.g., IL4 and IL-13) in adipose beiging (17–20). Data for the remaining genes are in Table S4 in Supplementary Material.

**Table 3:** Gene expression changed by cold after KDT501 treatment.

Gene^a^	Pre cold	Post cold	Fold change	*P*
			Post/pre	
ANGPTL4	1,136 ± 174	1,659 ± 163	1.46	0.001
CCL1	7 ± 1	12 ± 1	1.59	0.003
BCL2	456 ± 35	543 ± 45	1.19	0.004
CSF2	46 ± 4	53 ± 3	1.17	0.009
LIPE	225 ± 27	245 ± 26	1.09	0.018
CEBPA	6,498 ± 568	7,549 ± 504	1.16	0.019
PPARA	179 ± 13	192 ± 12	1.07	0.030
VEGFA	1,013 ± 86	1,150 ± 75	1.13	0.035
PPARGC1A	48 ± 6	57 ± 9	1.19	0.038
DIO2	41 ± 9	24 ± 5	0.58	0.049
TEK	263 ± 18	305 ± 24	1.16	0.051
MMP9	311 ± 117	210 ± 42	0.68	0.052
ABHD5	537 ± 65	597 ± 74	1.11	0.054
FABP3	219 ± 61	148 ± 13	0.68	0.065
ITGAM	291 ± 27	256 ± 19	0.88	0.068
ANGPTL1	288 ± 27	222 ± 14	0.77	0.075
PNPLA2	6,664 ± 648	7,129 ± 490	1.07	0.078
LPL	16,496 ± 2,584	18,240 ± 2,305	1.11	0.090
FGF2	1,019 ± 107	120 ± 91	1.10	0.092

*^a^SCWAT was isolated before and after the cold stimulus after KDT501 treatment, and gene expression was determined with the Nanostring nCounter system as described in the Section “Materials and Methods”*.

*The data are presented as means (nCounter Counts) ± SEM (*n* = 9; two-tailed, paired Student’s *t*-test)*.

**Figure 2:**
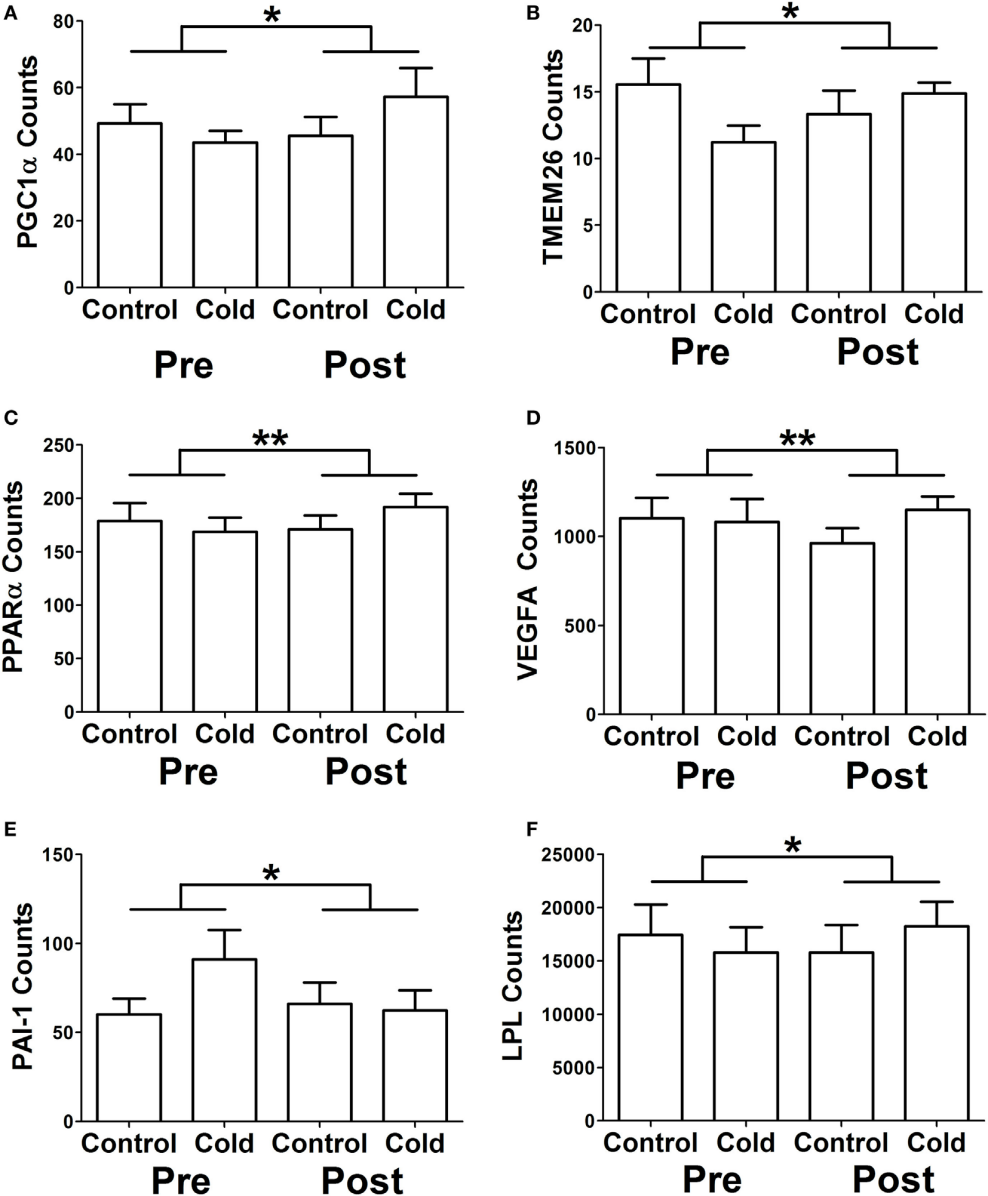
KDT501 treatment alters the subcutaneous white adipose tissue (SC WAT) transcriptional response to cold exposure. The Nanostring nCounter system was used to measure gene expression in SC WAT of subjects treated before and after 1 month KDT501 treatment. **A-F)** Genes that had a different response to the cold stimulus were identified as described in the Section “Materials and Methods.” Data represent the mean ± SEM (*n* = 9); data in all panels were analyzed by a paired, two tailed Student’s *t*-test of the change in gene expression by cold before and after KDT501 treatment (*P < 0.05; **P < 0.01).

We identified genes that had a differential response to cold before and after KDT501 treatment as described in methods (Table S5 in Supplementary Material). This analysis identified many of the same genes in Table 3 and some additional genes, providing further insight. Before KDT501 treatment, the gene expression of the thermogenic genes PGC1α, TMEM26, and PPARα decreased slightly after cold stimulation; after KDT501 treatment, the expression of these genes increased (Figures 2A–C; *P* < 0.05). VEGF, which induces beiging (14) and angiogenesis, was also increased by cold after KDT501 treatment (Figure 2D; *P* < 0.01). PAI-1 is regulated by TGFβ signaling, which inhibits PGC1α and energy expenditure (21); PAI-1 was increased by cold before KDT501, but did not increase after KDT 501 (Figure 2E; *P* < 0.05). Overall, this suggests that KDT501 promotes the expression of genes involved in lipolysis and fat catabolism in response to cold. In addition, LPL increased in response to cold after KDT501 treatment (Figure 2F).

### KDT501 Enhances β-Adrenergic Responses and Fatty Acid Oxidation in Adipocytes

**Figure 3:**
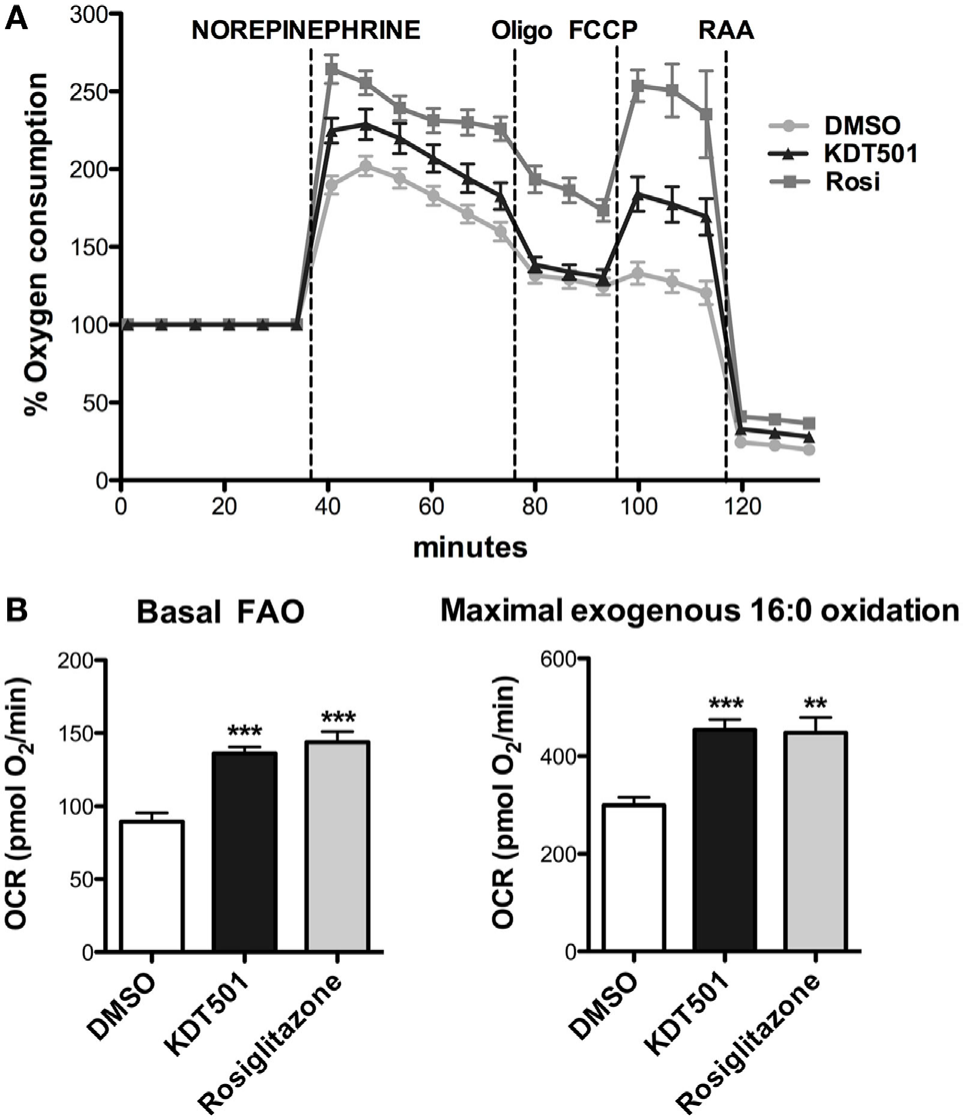
Adipocytes treated with KDT501 show a magnified response to norepinephrine and increased fatty acid oxidation rate. **(A)** Mouse primary brown adipocytes were treated for 16 h with DMSO (vehicle), KDT501 (10 µM), or rosiglitazone (2 µM). Oxygen consumption rate was measured on a Seahorse XFe96 as described in the Section “Materials and Methods.” **(B)** 3T3-L1 adipocytes were treated for 48 h with KDT501 (10 µM) or rosiglitazone (2 µM), and the rate of basal (endogenous) and maximal (exogenous palmitate added) fatty acid oxidation measured in real time using the XFe96. Data represent four biological replicates. Error bars indicate SD. **P < 0.01; ***P < 0.001 by one-way analysis of variance.

A potential explanation for the observation that KDT501 treatment enhances expression of thermogenic genes in response to cold is that KDT501 sensitizes adipocytes to β-adrenergic stimuli. To directly test this notion, fully differentiated mouse primary brown adipocytes were pretreated with KDT501 or rosiglitazone for 16 h, and their response to acute exposure to NE was then monitored in real time by measuring the rate of oxygen consumption in a Seahorse XFe instrument. As expected for brown adipocytes, NE exposure stimulated oxygen consumption in vehicle (DMSO)-treated cells (Figure 3A). Pretreatment with KDT501 robustly enhanced the response to NE (Figure 3A; KDT501 *P* < 0.001; rosiglitazone *P* < 0.001). Pretreatment with the positive control rosiglitazone had a similar effect. To expand these observations and determine the extent to which KDT501 treatment could also induce a more oxidative state in white adipocytes, we treated fully differentiated 3T3-L1 adipocytes with KDT501 for 48 h and determined the rate of fatty acid oxidation. KDT501 treatment significantly increased the endogenous, or basal (*P* < 0.001), and the maximal (*P* < 0.001) rate of exogenous fatty acid oxidation (Figure 3B), suggesting that KDT501 treatment boosts mitochondrial function. Together, these data show that KDT501 has direct effects on adipocytes, potentiating β-adrenergic signaling and fatty acid oxidation.

### Cold Treatment Stimulates Adiponectin Secretion

**Figure 4:**
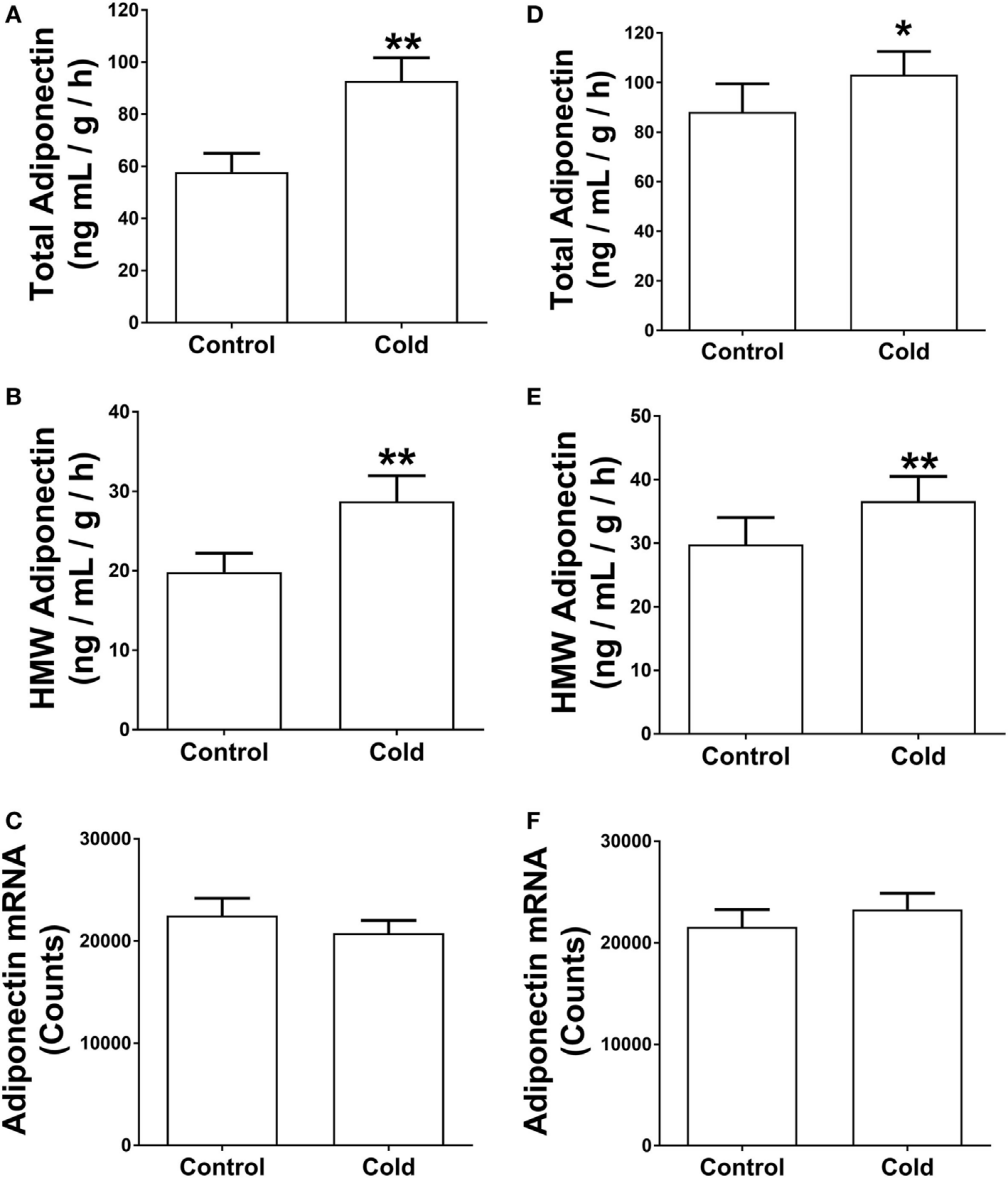
Cold induces total and high-molecular weight (HMW) adiponectin secretion by adipose tissue explants of obese, insulin-resistant subjects. Adipose tissue explants obtained before and after cold treatment (30 min) before and after KDT501 treatment were incubated in Dulbecco’s modified Eagles’ medium (DMEM) for 4-h at 37°C. **(A)** Total and **(B)** HMW adiponectin secretion in the DMEM were measured by ELISA and are expressed as the concentration/g adipose/h. **(C)** Adiponectin gene expression was measured in the corresponding adipose tissue biopsy with the Nanostring nCounter system as described in the Section “Materials and Methods.” **(D)** Total and **(E)** HMW adiponectin secretion in the DMEM were measured by ELISA. **(F)** Adiponectin gene expression was measured in the corresponding adipose tissue biopsy with the Nanostring nCounter system as described in the Section “Materials and Methods.” Data represent the mean ± SEM (*n* = 9); data in all panels were analyzed by a paired, two tailed Student’s *t*-test (*P < 0.05; **P < 0.01).

The analysis of gene expression and *in vitro* assays above suggest that KDT501 enhances β-adrenergic responses of adipocytes. Recently, a study by Komai and colleagues found that β- adrenergic signaling promotes adiponectin secretion (22). Since cold stimulates the sympathetic nervous system and β-adrenergic signaling, another potential mechanism for KDT501 stimulation of adiponectin secretion could involve this pathway. To explore this further, we measured adiponectin protein levels from the SC WAT explants and observed that cold-stimulated adiponectin secretion. Cold-stimulated total (Figure 4A; *P* < 0.01) and HMW adiponectin (Figure 4B; *P* < 0.01) secretion from the SC WAT explants before KDT501 treatment but did not affect adiponectin mRNA expression (Figure 4C). Cold also stimulated total (Figure 4D; *P* < 0.05) and HMW adiponectin (Figure 4E; *P* < 0.01) secretion from the SC WAT explants after KDT501 treatment but did not affect adiponectin mRNA expression (Figure 4F).

**Figure 5:**
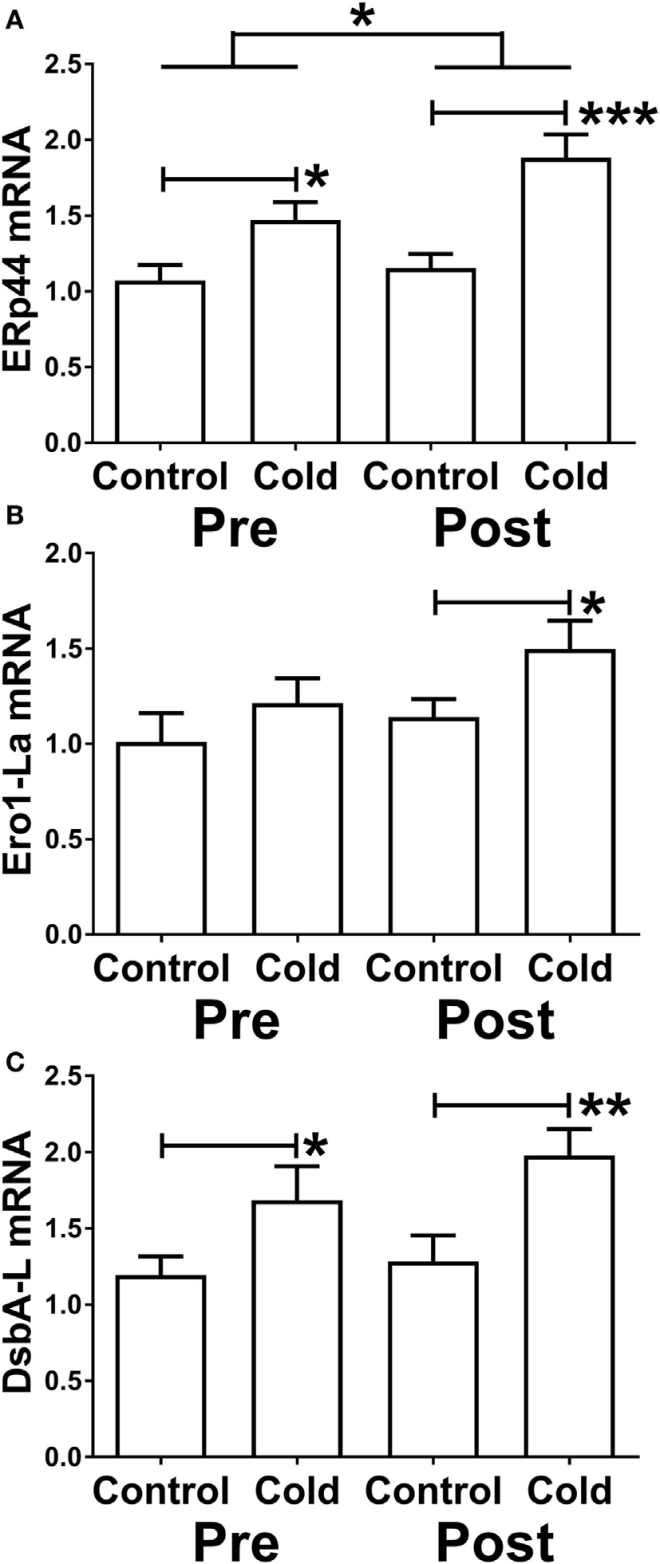
Effect of KDT501 and cold on the expression of genes that regulate adiponectin secretion and multimerization. **(A–C)** Gene expression was measured by real-time reverse transcriptase polymerase chain reaction as described in the Section “Materials and Methods.” Data represent the mean ± SEM (*n* = 9); data in all panels were analyzed by a repeated measures analysis of variance (*P < 0.05; **P < 0.01; ***P < 0.001). The data in all panels were also analyzed by a paired, two tailed Student’s *t*-test of the change in gene expression by cold before and after KDT501 treatment (ERp44: *P < 0.05).

Three proteins that promote adiponectin secretion have been identified and could thus act posttranscriptionally to increase adiponectin secretion. Because of the increase in adiponectin secretion without any change in mRNA level, we therefore determined whether KDT501 or cold affected the expression of these by real-time RT-PCR (primer sequences are in Table 4). KDT501 treatment alone did not affect the mRNA expression of any of these; however, all three were induced by cold. ERp44 was induced by cold (Figure 5A; pre: *P* < 0.05; post: *P* < 0.001), and KDT501 enhanced this effect (Figure 5A; *P* < 0.05). Ero-La was induced by cold after KDT501 (Figure 5B; *P* < 0.05). DsbA-L was induced by cold before and after KDT501 treatment (Figure 5C; pre: *P* < 0.05; post: *P* < 0.01).

**Table 4:** Real-time reverse transcriptase polymerase chain reaction primers.

Gene	Forward	Reverse
ACTB	GAG CAC AGA GCC TCG CCT TT	CGC GGC GAT ATC ATC ATC CAT
PP1A	CCC ACC GTG TTC TTC GAC AT	GCT GTC TTT GGG ACC TTG TCT
PP1B	AAG TCA CCG TCA AGG TGT ATT TT	TGC TGT TTT TGT AGC CAA ATC CT
TBP	CCC GAA ACG CCG AAT ATA ATC C	AAT CAG TGC CGT GGT TCG TG
TUBB	ACC AAC CTA CGG GGA TCT GAA	TTG ACT GCC AAC TTG CGG A
UBC 9	CTG GAA GAT GGT CGT ACC CTG	GGT CTT GCC AGT GAG TGT CT
ERp44	AGC CCA GAG ATA CAG GAT AAG C	GTT GCC TGA TGT AAT CTG CCA
ERO1L	GGC TGG GGA TTC TTG TTT GG	AGT AAC CAC TAA CCT GGC AGA
DsbA-L	TCT GGA AAA GAT CGC AAC GC	GCC CAA AGG CTC CGT ATC TG

### Summary

In summary, KDT501 treatment had a beneficial effect on SC WAT of obese human research participants. KDT501 increased adiponectin secretion by a posttranscriptional mechanism and altered the transcriptional response of human adipose tissue to cold stimulation, inducing the expression of many genes involved in thermogenesis and lipolysis. Consistent with these observations, the ability of KDT501 to enhance the response of cultured adipocytes to β-adrenergic stimuli and increase mitochondrial function provides a potential mechanism of action.

## Discussion

There is a need to develop new therapies to combat obesity and obesity-related disorders such as metabolic syndrome, non-alcoholic fatty liver disease and steatohepatitis, and the lipodystrophy of PCOS. We previously reported that KDT501 treatment of obese, insulin-resistant subjects for 28 days resulted in an increase in plasma adiponectin and HMW adiponectin (2). In this study, we found that SC WAT from these obese, non-diabetic human subjects is a target of KDT501 treatment and investigated the mechanistic basis of its action. KDT501 stimulates total and HMW adiponectin protein secretion from SC WAT explants, and this may be related to the ability of KDT501 to enhance mitochondrial function, which was revealed in experiments using cultured adipocytes. Furthermore, additional analysis uncovered additional effects of KDT501 on SC WAT. Multiplex analysis of gene expression revealed that KDT501 enhanced expression of PGC1α, PPARα, and the beige cell marker TMEM26 in response to cold. These findings are consistent with the ability of KDT501 to enhance β-adrenergic responses in primary brown adipocytes.

Adiponectin exists as a heterogeneous mix of multimers, and plasma HMW adiponectin levels are negatively correlated with various aspects of metabolic syndrome, including insulin resistance (4). Following the transcription of the adiponectin 30 kDa monomer, considerable posttranslational processing and assembly of the HMW multimer occurs, leaving multiple possibilities for regulation. The inability of KDT501 to increase transcription of the adiponectin gene indicates that KDT501 induces adiponectin secretion and multimerization posttranscriptionally. Multimerization of adiponectin into an HMW form and its secretion are complex processes regulated by ER stress, mitochondrial dysfunction, autophagy, and proteins such as ERp44, Ero1-Lα, and DsbA-L (23–26). Our studies demonstrating that KDT501 enhances fatty acid oxidation in 3T3-L1 adipocytes suggest that KDT501 has beneficial effects on mitochondrial function. Mitochondrial function in adipose tissue is impaired in obesity, and recent studies suggest that impaired mitochondria function reduces adiponectin secretion (27–29). Therefore, the ability of KDT501 to increase adiponectin secretion may be through its ability to enhance mitochondrial function, as was revealed by our *in vitro* assays. Another possible mechanism for the increase in adiponectin secretion induced by KDT501 could be that KDT501 sensitizes adipocytes to β-adrenergic agonists, as was revealed by our analysis of SC WAT gene expression and *in vitro* assays. Recent studies have demonstrated that β-adrenergic signaling pathways promote adiponectin secretion (22). We found that cold stimulates adiponectin secretion *in vivo*, suggesting that stimulation of β-adrenergic signaling increases adiponectin secretion *in vivo* in obese subjects. Thus, the ability of KDT501 to enhance β-adrenergic signaling may also contribute to the increased adiponectin secretion.

Pioglitazone has also been shown to induce adiponectin secretion by posttranscriptional mechanisms (30), and PPARγ agonists are known for their powerful effects on increasing adiponectin gene expression (31). However, data from this and other studies are not consistent with KDT501 directly stimulating PPARγ transcriptional activity. *In vitro*, KDT501 has been reported to have pleotropic effects in adipocytes, including suppression of NFκB signaling (1), and, perhaps, a weak PPARγ agonist effect. However, in this study, we found no evidence of KDT501 PPARγ agonism in humans. Direct PPARγ target genes such as adiponectin, LPL, CD36, PNPLA2, and FABP4 were not induced by KDT501 treatment (Table S2 in Supplementary Material). Suppression of inflammation would also be predicted to promote adiponectin gene expression, but numerous inflammatory cytokine and immune cell markers were measured and not affected by KDT501. Thus, it will be important to perform additional studies to determine the mechanism by which KDT501 exerts changes in adiponectin circulating levels. Our *in vitro* studies suggest that an improvement in mitochondrial function may be an important part of the mechanism.

The data in this report are consistent with the increase in plasma adiponectin in these research participants reported earlier (2). Adipose tissue explants from these same subjects secreted more total and HMW adiponectin after KDT501 treatment. Although adiponectin levels in plasma were increased, our clinical study did not reveal changes in glucose metabolism or insulin sensitivity (2). These subjects were only treated with KDT501 for 28 days, and it is possible that longer treatment or higher blood levels of KDT501 are necessary to realize the full beneficial effects of its ability to increase adiponectin.

Despite increasing the levels of adiponectin, which is an anti-inflammatory adipokine, KDT501 had minimal effects on gene expression in the absence of a cold stimulus. One explanation for the lack of effect on gene expression is that the short-term treatment did not cause weight loss (2), change % body fat, or change resting energy expenditure. KDT501 treatment remodeled the transcriptional response of adipose tissue to cold and increased expression of genes involved in lipolysis and thermogenesis. We have recently reported that lean, healthy subjects respond to the same cold stimulus used in this study with a significant increase in expression of PGC1α and TMEM26 at the RNA level (13). The induction of PGC1α and TMEM26 in response to cold after KDT501 treatment seen here was lower than that measured in the lean subjects of our earlier study (13). Nonetheless, this indicates a beneficial effect of KDT501 on SC WAT, for PGC1α expression in white adipose tissue promotes insulin sensitivity (32). It is also important to note that this study was done entirely in the summer. We have previously documented seasonal effects on thermogenic gene expression in humans. Baseline expression of thermogenic genes in lean subjects is lower in the summer, and we saw greater changes in response to a cold stimulus in the summer (13). These findings indicate reduced β-adrenergic signaling in humans in the summer, which may explain why greater changes in PGC1α and TMEM26 expression after KDT501 treatment were not seen in the present obese subjects. KDT501 robustly enhanced NE-stimulated cellular respiration in primary brown adipocytes, suggesting a mechanism for the increase in PGC1α and TMEM26 expression upon cold exposure *in vivo*: KDT501 sensitizes adipose tissue to β-adrenergic signaling. This mechanism may contribute to the weight loss observed in KDT501-treated mice, studies that were performed at room temperature, below thermoneutrality for mice (1).

ANGPTL4 expression was highly induced by cold after KDT501 treatment. Since ANGPTL4 can be induced by free fatty acids acting through PPARδ (33), this may be a sign of increased lipolysis. Interestingly, ANGPTL4 itself contributes to lipolysis by stimulating adipocyte cAMP levels (34, 35). Recent studies in mice indicate that ANGPTL4 is induced in WAT in response to cold to inhibit LPL and thus favor the delivery of lipids to BAT (36). KDT501 appears to restore this tissue-specific regulation of ANGPTL4 by cold in obese subjects. PGC1α was also induced by cold after KDT501 treatment along with a number of other genes involved in lipid catabolism, including PPARα. Notably, UCP1 expression was not induced. This finding may be ascribed to the fact that cold-stimulated induction of UCP1 is blunted by chronic inflammation in obese subjects (13), and KDT501 did not reduce SC WAT inflammation, as evidenced by a lack of change in expression of multiple inflammatory genes. It is also possible that the timing of the biopsies, 4 h after the 30 min cold stimulus, may not have been suited to capture cold-induced changes in UCP1 mRNA.

Taken together, the results of this initial study reveal that SC WAT is a physiologic target of KDT501. KDT501 acts by at least two mechanisms. It promotes adiponectin secretion by a posttranscriptional mechanism that may be related to its ability to improve mitochondrial function. KDT501 also enhances thermogenic gene expression upon cold exposure, a property that may be due to its ability to sensitize cultured adipocytes to β-adrenergic stimuli. Longer exposure to KDT501 may have beneficial effects on adipose tissue dysfunction implicated in conditions such as T2D, PCOS, and NASH in humans.

## Ethics Statement

All subjects gave informed consent, and the protocols were approved by the Institutional Review Board at the University of Kentucky.

## Author Contributions

PK, ES, and BF designed the experiments, analyzed data, and wrote the manuscript. BZ, BK, and CG performed the experiments. PW analyzed data. NG, RS, and JB edited the manuscript. PK is the guarantor of this work, has full access to all of this data, and takes responsibility for it integrity and the accuracy of its analysis.

## Conflict of Interest Statement

This study was funded by Kindex Pharmaceuticals (Seattle, WA).
